# Giga-Pixel Lensfree Holographic Microscopy and Tomography Using Color Image Sensors

**DOI:** 10.1371/journal.pone.0045044

**Published:** 2012-09-12

**Authors:** Serhan O. Isikman, Alon Greenbaum, Wei Luo, Ahmet F. Coskun, Aydogan Ozcan

**Affiliations:** 1 Electrical Engineering Department, University of California Los Angeles, Los Angeles, California, United States of America; 2 Bioengineering Department, University of California Los Angeles, Los Angeles, California, United States of America; 3 California NanoSystems Institute, University of California Los Angeles, Los Angeles, California, United States of America; 4 Department of Surgery, School of Medicine, University of California Los Angeles, Los Angeles, California, United States of America; Virginia Tech, United States of America

## Abstract

We report Giga-pixel lensfree holographic microscopy and tomography using color sensor-arrays such as CMOS imagers that exhibit Bayer color filter patterns. Without physically removing these color filters coated on the sensor chip, we synthesize pixel super-resolved lensfree holograms, which are then reconstructed to achieve ∼350 nm lateral resolution, corresponding to a numerical aperture of ∼0.8, across a field-of-view of ∼20.5 mm^2^. This constitutes a digital image with ∼0.7 Billion effective pixels in both amplitude and phase channels (i.e., ∼1.4 Giga-pixels total). Furthermore, by changing the illumination angle (e.g., ±50°) and scanning a partially-coherent light source across two orthogonal axes, super-resolved images of the same specimen from different viewing angles are created, which are then digitally combined to synthesize tomographic images of the object. Using this dual-axis lensfree tomographic imager running on a color sensor-chip, we achieve a 3D spatial resolution of ∼0.35 µm×0.35 µm×∼2 µm, in x, y and z, respectively, creating an effective voxel size of ∼0.03 µm^3^ across a sample volume of ∼5 mm^3^, which is equivalent to >150 Billion voxels. We demonstrate the proof-of-concept of this lensfree optical tomographic microscopy platform on a color CMOS image sensor by creating tomograms of micro-particles as well as a wild-type *C. elegans* nematode.

## Introduction

**Figure 1:**
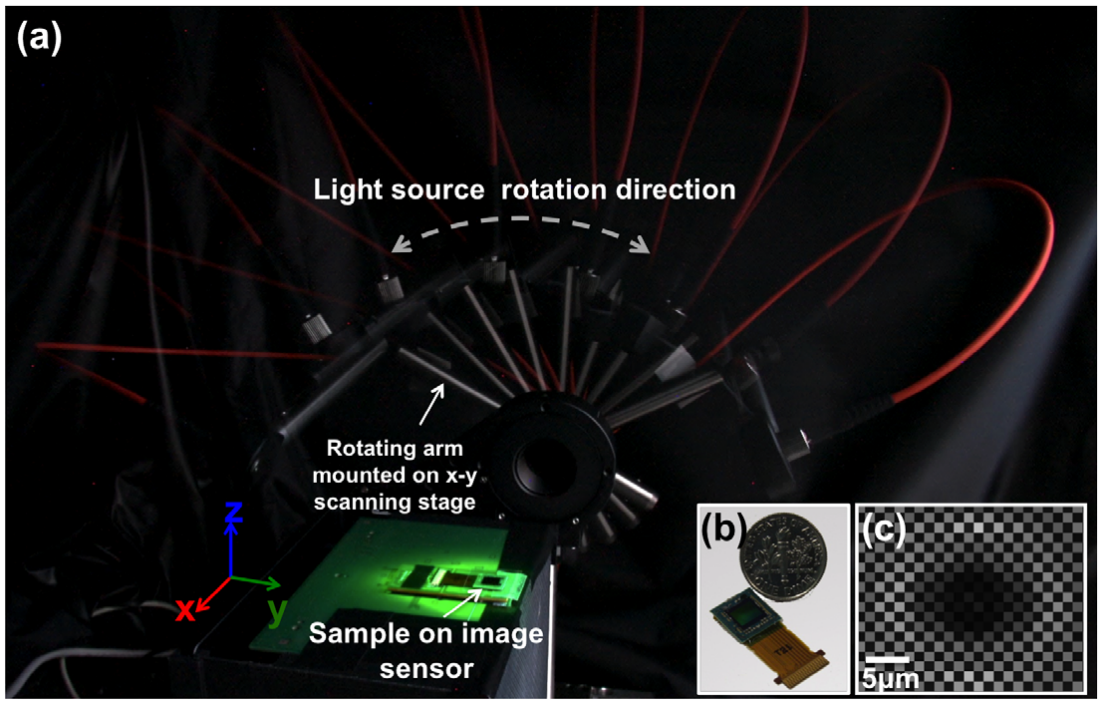
Giga-Pixel Lensfree Holographic Microscopy and Tomography setup. (a) Shows a multi-exposure photograph of the lensfree optical tomography setup employing a colour image sensor. An optical fibre attached to a rotation stage delivers partially-coherent light to the sample, placed on the sensor, at different angles. The light source is also translated laterally using scanning stages to perform pixel super-resolution at each illumination angle. (b) Shows the photograph of the colour image sensor chip. The protective glass, colour filters and the microlens-array on the active area of the sensor remain intact. (c) Shows a cropped lensfree hologram image to demonstrate the Bayer colour filter pattern.

**Figure 2:**
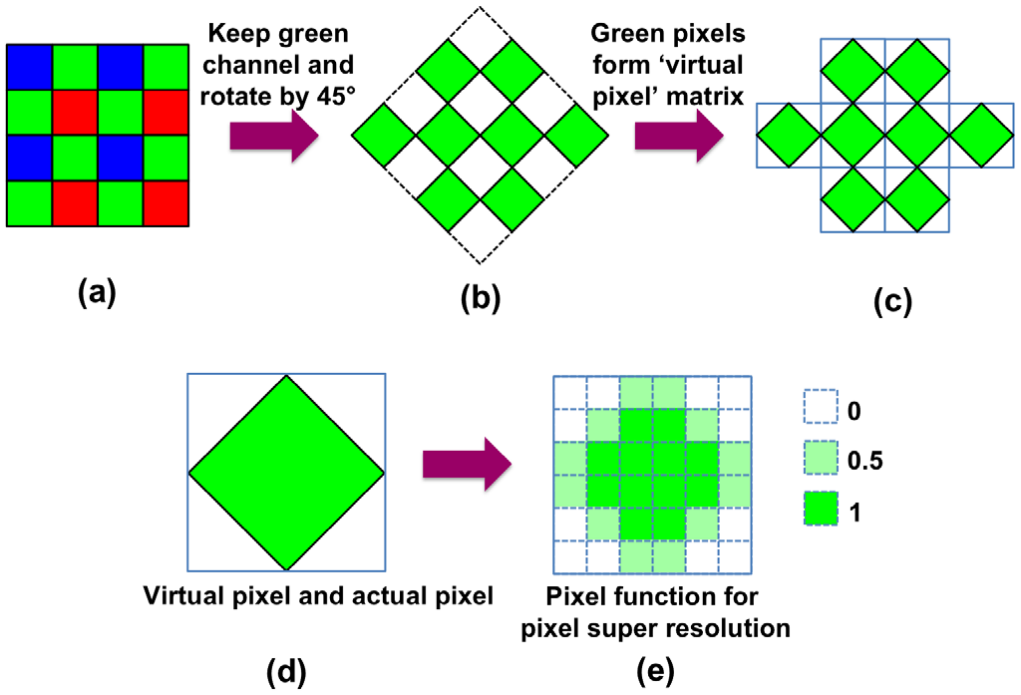
Overview of the method for obtaining grayscale holograms using color imaging sensors. Illustrates the method used to obtain a grayscale hologram using a colour sensor-array. (a) First, the green pixels are selected from the raw output image of the sensor. (b) The resultant data array is rotated 45° to obtain virtual pixels. (c) Virtual pixel-array is obtained from the rotated array of green pixels in (b). (d) Shows a single “virtual pixel”, which is a unit element of the pixel-array shown in (c). (e) The weighted pixel function to be used in pixel super-resolution is obtained, representing the 2D spatial map of light collection within a virtual pixel.

Color opto-electronic sensors such as CMOS imagers that exhibit Bayer patterns (composed of one Red, two Green and one Blue pixels) form the main stream detector-arrays employed in digital electronic devices, including cell phones, webcams and digital cameras, with a sales volume of >5 billion per year [1]. The use of such cost-effective yet powerful imaging components has been an emerging theme for various applications including point-of-care microscopy and sensing [2]–[8]. Along the same lines, lensfree computational imaging techniques [9]–[25] also demand state-of-the-art sensor-arrays that employ smaller pixel sizes as well as larger pixel counts (for increased imaging field-of-view). By making use of such advanced image sensors, lensfree microscopy can provide sub-micron spatial resolution over large imaging areas, within a rather compact and cost-effective design, which is particularly suitable for telemedicine needs and lab-on-a-chip platforms. While consumer electronics has been the driving motivation to create such sensor chips [26], an important obstacle for lensfree holographic imaging has been the Bayer filter pattern (i.e., Red, Green and Blue filters) installed on color sensor-chips. Since holographic microscopy techniques typically employ quasi-monochromatic light sources [12]–[23], [27]–[36] (with spectral bandwidths of ∼1–30 nm), the pixels coated with different color filters respond differently to incident light. As a result, the Bayer color filter pattern installed on the imaging area can introduce undesired artefacts. While color sensors have been successfully employed in phase-shifting digital holography schemes to achieve color imaging with multiple lasers [27], such sensors unfortunately create artefacts in lensfree on-chip holography, particularly impeding the use of pixel super-resolution algorithms [19], [20], [37], [38]. This problem can potentially be mitigated by e.g., physical removal of such color filters placed above the active region of each pixel. While feasible, physical removal of such color filters is costly to implement in high-volumes, and could alter the optimized design of pixel structures. Another option is to use monochrome version of the same sensor-chip of interest, which, however, is not often released by CMOS manufacturers since the main application areas, i.e., cell phones, and webcams, strictly demand color sensor-arrays. To better handle this challenge, here we introduce a new computational approach to utilize color sensor-arrays in lensfree microscopy and tomography for achieving Giga-pixel imaging on a chip. Our imaging setup (Fig. 1.a) is based on lensfree on-chip holography [5], [12]–[23], where a partially coherent light source is used to illuminate a sample that is placed on the top of a sensor array, to record digital in-line holograms of objects with unit fringe magnification over a large field-of-view (see the Methods section). In this work, we specifically employed a color CMOS sensor chip (Fig. 1.b) that has a pixel size of 1.12 µm and an active area of 20.5 mm^2^. Without physically removing the color filters installed on a sensor chip, based on a new reconstruction approach, as illustrated in Fig. 2, we synthesize pixel super-resolved lensfree holograms of specimen using 45° rotated green pixel functions (refer to Methods Section for details). These super-resolved holograms are then reconstructed to achieve ∼350 nm lateral resolution, corresponding to a numerical aperture (NA) of ∼0.8, across a field-of-view of ∼20.5 mm^2^. This constitutes a lensfree digital image with ∼0.7 Giga-pixels effectively, in both amplitude and phase channels, i.e., ∼1.4 Giga-pixels in total. It should also be noted that the use of oil immersion techniques [39] can further improve the lateral resolution, permitting numerical apertures reaching up to e.g., ∼0.9, as recently demonstrated in our work [40]. In addition, by changing the illumination angle over ±50 degrees, pixel super-resolved images of the same object from different viewing angles can be created and digitally combined to form lensfree tomograms of the object (see Methods Section) [23], [41]. The architectural simplicity of this platform also permits scanning the light source across two orthogonal axes to obtain additional perspective images of the sample, and further improve tomographic imaging quality. With this dual-axis lensfree tomographic imager running on a color CMOS chip, we achieved a spatial resolution of ∼0.35 µm×0.35 µm×∼2 µm, in the x, y and z directions, respectively. These results create an effective voxel size of ∼0.03 µm^3^ across a sample volume of ∼5 mm^3^, which is equivalent to >150 Billion voxels.

Compared to our earlier lensfree on-chip tomography platform [23], which utilized relatively older generation monochrome sensor-chips, these current results constitute more than an order of magnitude increase in our voxel density, significantly improving the 3D space-bandwidth product of our lensfree imaging platform. We validated the performance of this on-chip tomographic microscope by imaging micro-particles and a wild-type *C. elegans* nematode.

## Results

**Figure 3:**
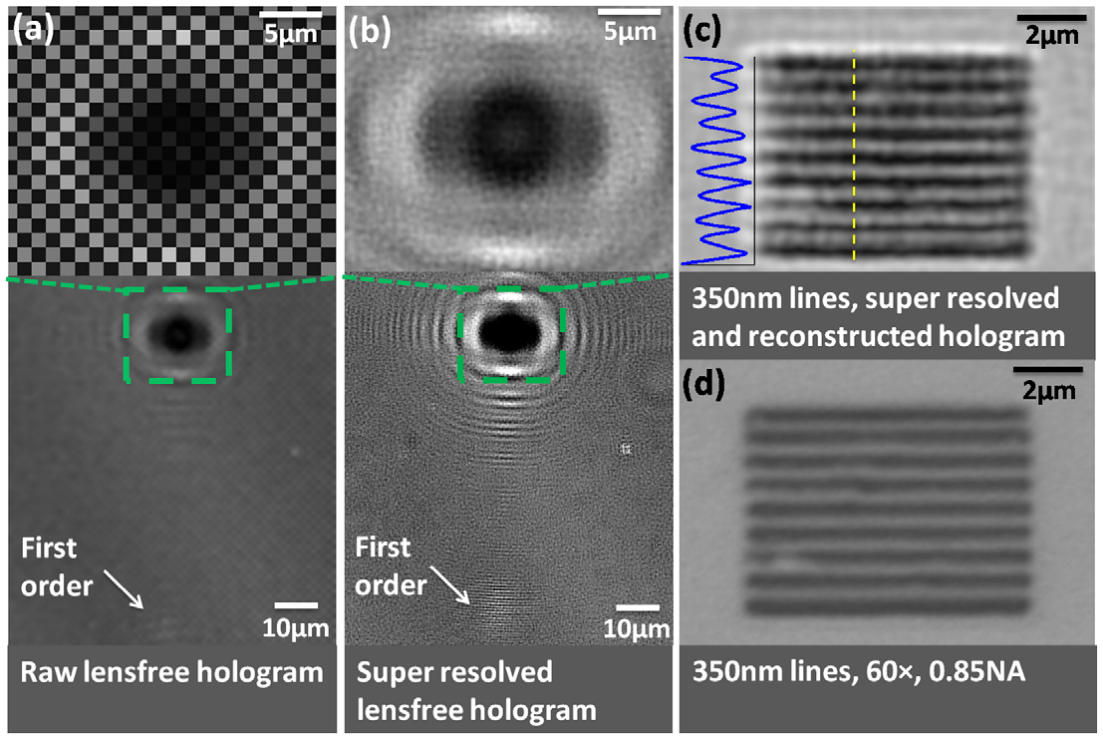
Lensfree imaging results demonstrating 0.35 µm half-pitch lateral resolution. (a) Shows a cropped raw lensfree hologram of a grating structure etched on glass. The fringes on the first diffraction order (indicated by the arrow) cannot be resolved due to under-sampling. (b) Shows a pixel super-resolved (SR) hologram of the same grating. The fringes on the first diffraction order are now resolved. (c) Shows the lensfree image obtained by reconstructing the SR hologram, demonstrating that the grating lines can be clearly resolved. (d) Shows a bright-field microscope image (60×, 0.85-NA) of the same grating for visual comparison. Note that the contrast in (a) and (b) was enhanced for illustration of fine holographic oscillations.

To quantify the lateral resolution of our lensfree holographic set-up based on color CMOS sensor-arrays and validate our modified pixel super-resolution method (Fig. 2), we imaged a grating with 350 nm lines etched on glass (i.e., 700 nm period), which was fabricated using focused ion beam (FIB) milling. Fig 3.a shows the raw lensfree hologram of the grating, cropped from a large FOV (∼20.5 mm^2^). The first diffraction order can be seen in the holographic image; although, the fringes for this order could not be resolved due to spatial under-sampling. The zoomed inset (top image) in Fig. 3.a also illustrates the presence of the Bayer pattern artefact in this image.

Based on our modified pixel-super resolution approach (see the Methods Section and Fig. 2), both the Bayer pattern of the color sensor-array and the under-sampling related artefacts were mitigated using 64 (8×8 grid) sub-pixel shifted lower-resolution in-line holograms (see Fig. 3.b). Consequently, the grating object with a half-period of 350 nm could be reconstructed, as shown in Fig. 3.c. A conventional bright-field microscope image (60×, 0.85-NA) of the same grating is also provided for comparison (Fig. 3.d). This result demonstrates the lateral resolving power of our lensfree on-chip microscopy system based on a color CMOS imager, achieving a numerical aperture (NA) of ∼0.8.

**Figure 4:**
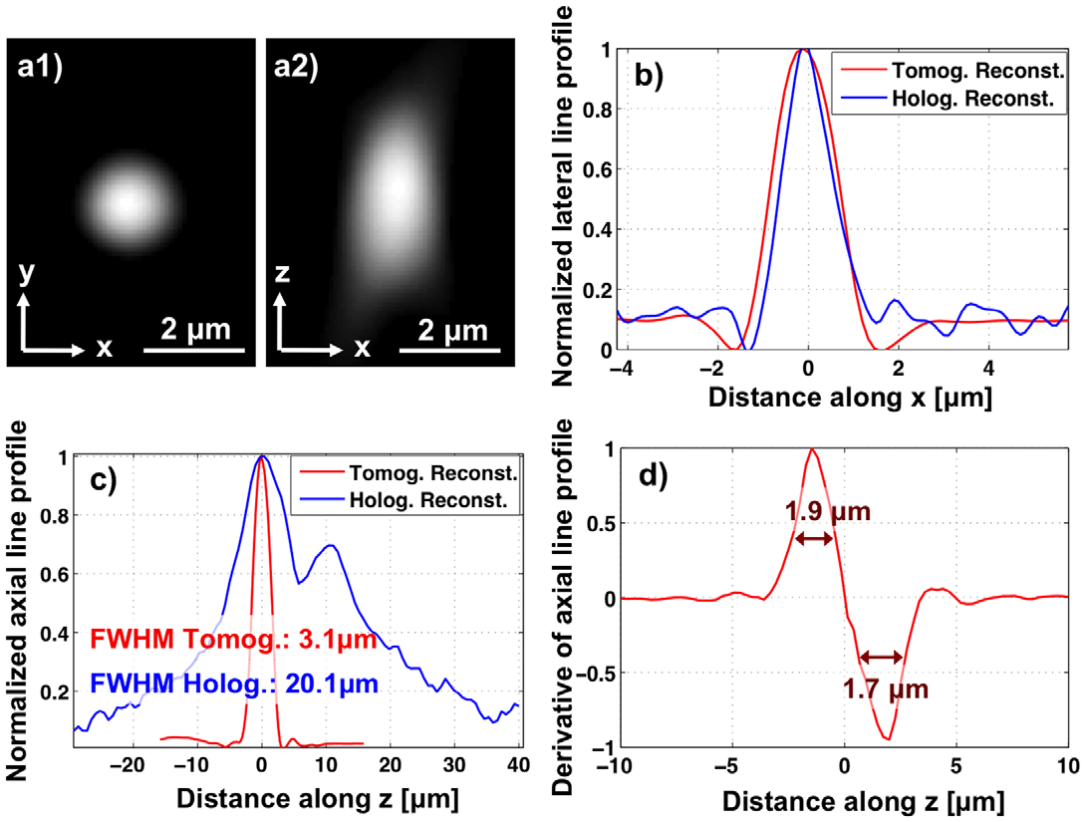
Lensfree imaging results demontrating ∼2 µm axial resolution. (a1) Shows a tomogram in the x-y plane (lateral slice image) through the centre of a micro-particle having a diameter of 2 µm. (a2) Shows a tomogram in the x-z plane (ortho-slice image). The axial elongation is a manifestation of the limited angular range used in our tomographic reconstruction. (b) Shows line profiles along the x direction through the centre of the micro-particle’s tomographic (red curve) and holographic images (blue curve). (c) Shows axial line profiles through the centre of the micro-particle’s tomographic (red curve) and holographic images (blue curve). The significant improvement in axial resolution can be observed, as the FWHM of the axial line profile after tomographic reconstruction is much smaller. (d) Shows the derivative of the tomographic axial line profile in (c). Based on this edge response result, an axial resolution of ∼2 µm can be estimated.

In order to estimate the axial resolution of our platform, we imaged spherical micro-particles that have a diameter of 2 µm. These melamine micro-particles (refractive index: 1.68) were randomly distributed in a chamber filled with an optical adhesive gel (refractive index: 1.56). The chamber had a thickness of ∼80 µm. The sample was placed on the color CMOS sensor with ∼100 µm distance to its active area for lensfree tomographic imaging. Figures 4.a1-a2 show the cross-sectional images of an arbitrary micro-particle obtained by tomographic reconstruction (see the Methods Section). While the bead appears circular in the x-y plane passing through its centre, the x-z plane reveals that the bead appears axially elongated (along the z direction). This is a manifestation of the limited angular range that is used in our lensfree optical tomography set-up, as a result of which the axial resolution is lower than the lateral one [23], [42], [43]. In order to estimate our axial resolution, we plotted the line profile through the centre of the bead (along the z direction), and measured its full-width-at-half-maximum (FWHM) value as ∼3.1 µm (Fig. 4.c). Moreover, we also calculated the one-dimensional spatial derivative of this axial line profile, which can be used to estimate the edge sharpness of these tomograms [44], [45]. Based on the FWHM values of this spatial derivative plot shown in Fig. 4.d, the axial resolution can be estimated to be ∼2 µm. Due to the relatively lower axial resolution of in-line holography, merely reconstructing a single in-line hologram of the same micro-particle does not provide the same level of axial resolution. As shown in Fig. 4.c, the axial line profile obtained by reconstructing a single vertical hologram at different depths exhibits an FWHM value of ∼20 µm. Therefore, it can be concluded that the multi-angle tomographic imaging approach provides a significant improvement on the sectioning ability of lensfree on-chip microscopy. It is important to note that with a single data acquisition step, a large imaging volume having an area of e.g., ∼20.5 mm^2^ and a height of e.g., 0.3 mm can routinely be probed [23], while the depth-of-field can be further increased to >1 mm at the cost of reduced spatial resolution.

To further demonstrate tomographic microscopy over a large FOV, supplementary Movie S1 shows imaging of multiple regions-of-interest (total imaging area ∼20.5 mm^2^) from the same chamber that contains randomly distributed particles with a diameter of 2 µm. It should also be noted that the tomogram field-of-view effectively shrinks at increasing sample-to-sensor distances since the lensfree holograms of objects close to edges of the sensor do not fall on the active area of the sensor at large illumination angles. An effective imaging volume of ∼5 mm^3^ can be calculated for a cut pyramid-shaped volume over which the aforementioned spatial resolution can be maintained.

**Figure 5:**
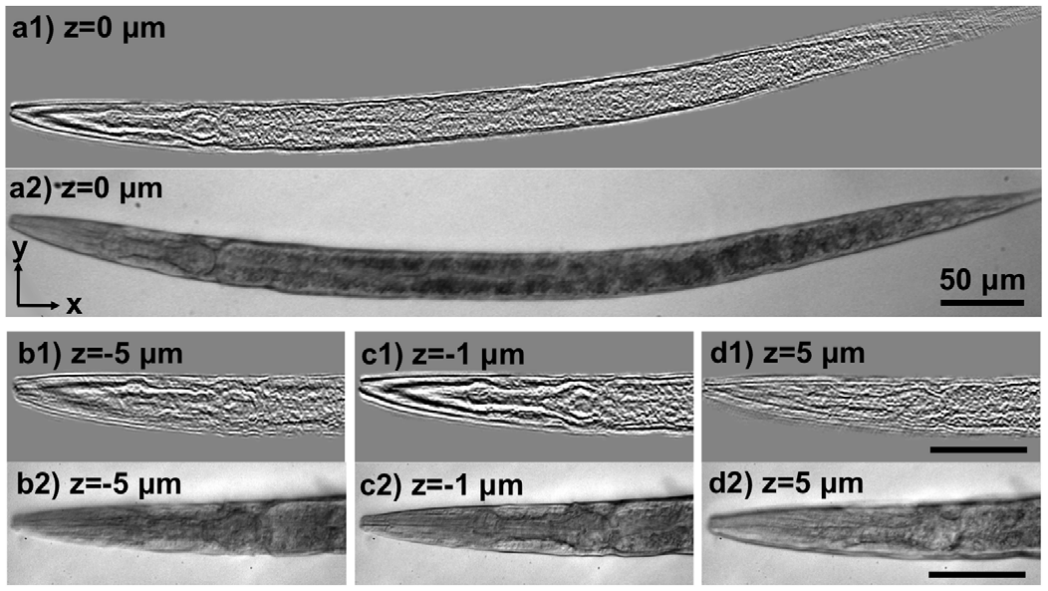
Gigapixel lensfree tomographic imaging of *C. elegans* **on a chip.** (a1) Shows the tomogram of a *C. elegans* nematode at z = 0 µm plane. (a2) Shows a bright-field microscope image (20×, 0.4-NA) showing the entire worm for qualitative visual comparison. (b1, c1, d1) Show tomograms at different depth layers through the pharynx of the worm. (b2, c2, d2) Show bright-field microscope images (60×, 0.85-NA) for the same depth layers as in (b1, c1, d1) for visual comparison. All scale bars: 50 µm.

To evaluate the performance of color sensor-array based lensfree optical tomography in biomedical applications, we also imaged a *C. elegans* worm. Fig. 5 shows three different slice images through the worm along with conventional bright-field microscope images (60×, 0.85-NA) for qualitative comparison. It can be observed that the *pharynx* of the worm is in-focus in the tomogram at z = 0 µm, and the *metacorpus* of the worm mostly disappears in the slices away from the centre, which can also be confirmed by the corresponding microscope images. Furthermore, the tomograms reveal that the pharyngeal tube of the worm is widest at z = 0 µm, and gets narrower at the outer slices, which is expected due to its cylindrical structure. It should be noted that a single axis tomography scheme was used in obtaining these results, where the illumination angle was varied along the direction orthogonal to the length of the worm, since including the other axis would not improve the image quality due to the elongated shape of the worm [23].

## Discussion

Using the state-of-the-art ‘color’ image sensors, lensfree optical tomography can now achieve Giga-pixel imaging with ∼350 nm lateral resolution (corresponding to an NA of ∼0.8) and ∼2 µm axial resolution, which results in an order of magnitude increased voxel density, i.e., space-bandwidth product, compared to what was previously achieved for on-chip tomography [23]. Owing to its large imaging area of 20.5 mm^2^ and long depth-of-field (DOF) of ∼0.3 mm, a large sample volume of ∼5 mm^3^ can be probed with >150 Billion voxels, which can be useful for wide-field imaging applications in lab-on-a-chip platforms. Here, we should also emphasize that Giga-pixel microscopy in the context of lensfree on-chip imaging refers to the *effective* number of digital pixels in the reconstructed image, with the assumption that 2 pixels define the minimum resolvable feature size. In this scheme, pixel super-resolution techniques permit achieving Giga-pixel imaging, which is different than the recent Giga-pixel photography work [46].

The use of a color sensor-array is an important step forward in lensfree holographic on-chip imaging. The rapid advances in imaging sensors are mainly driven by consumer electronics industry toward developing higher-resolution color sensor chips with larger active areas. Therefore, their incorporation in lensfree on-chip imaging platforms is critical such that off-the-shelf sensors can be cost-effectively integrated with compact lensfree microscopes to achieve immediate enhancement of image quality as better sensors become available. The results presented in this work demonstrate this trend, where an order of magnitude improvement in space-bandwidth product is achieved using a color CMOS imager and a modified pixel super-resolution algorithm that overcomes the limitations posed by the Bayer filters installed on sensor chips. It is important to note that even though our experimental results are obtained using the green pixels only, the illumination wavelength is not restricted to only ∼530 nm, as the spectral response of the green color filters is rather broad (i.e., FWHM ∼115 nm). Therefore using different illumination wavelengths is also possible, without any modification to the presented image processing technique or the experimental set-up.

The ability to image a large DOF is another important advantage that significantly enhances the imaging throughput. The key enabler for this is the in-line holographic recording scheme that permits digital focusing to an arbitrary depth of interest. Although the highest resolution is achieved when the sample-to-sensor distance is <0.3 mm, an extended DOF of e.g., 4–5 mm can be imaged at the cost of reduced spatial resolution due to lower detection SNR at increased heights above the sensor. It is important to note, however, that this extended DOF does not correspond to the thickness of a continuous sample that can be axially sectioned. For lensfree optical tomography to provide a decent image quality, the reconstructed images should represent projections, i.e. line integrals along the illumination direction, of a certain property of the object such as its phase, absorption or scattering potential functions. Currently, we use the amplitude of the reconstructed lensfree images, with the assumption that they represent the projections of the scattering strength of the object. The quality of reconstructions depends on the validity of this assumption. Thick objects, e.g. >50 µm, violate this assumption for two main reasons. First, the depth-of-focus of a reconstructed image will not be large enough such that all parts of such a thick object can contribute equally to the reconstructed image, as some parts of the object will be defocused. This limitation, however, can be partially mitigated by using a diffraction tomography approach [45]–[50], or by reconstructing each in-line hologram at different depths to estimate the correct weighting factors of slices at various depths at the cost of increased computational complexity. Second, and more importantly, thicker samples will strongly scatter the incoming photons. If light transmission is dominated by multiple scattering, as is the case when the thickness of the object exceeds the mean free path of photons through a turbid medium [51], a line-integral relationship between the reconstructed image and the structure of the object cannot be maintained. In this case, the filtered back-projection algorithm (see the Methods Section) will not provide accurate results and exhibit aberrations. Therefore, lensfree optical tomography appears to be particularly suitable for high-throughput imaging of cells and micro-organisms that are distributed within a thick chamber (e.g. ∼1–5 mm), rather than for imaging thick and optically dense specimen such as tissue slides. This limitation, however, is common to ‘all’ the existing on-chip imaging modalities, regardless of their operation principles.

## Methods

### Lensfree Optical Tomography Setup

A partially coherent light source (Xenon lamp attached to a monochromator), coupled to a multi-mode optical fiber (core diameter: 105 µm), is attached to a motorized rotation stage, which is also mounted on a scanning stage (see Fig. 1.a). For illumination, we used a centre wavelength of 530 nm with a spectral width of ∼3 nm. Although, in the current setup, 3 nm bandwidth was achieved using a monochromator for experimental flexibility, a light-emitting diode (LED) interfaced with a simple interference filter could also be utilized. The use of an interference filter does not significantly increase the cost and complexity of the system toward field-portable microscopy, since a filter with a rather small area (e.g. <1 mm×1 mm) placed right after the LED would be sufficient as demonstrated in our earlier work [21]. A color CMOS image sensor with a pixel pitch of 1.12 µm is employed (Fig. 1.b). The protective glass, the micro-lens array and the color filters on the chip remained intact. In this configuration the sample is placed with typically <300 µm distance to the active area. The rotation stage is used to sequentially illuminate the sample from different angles within a range of ±50°. To achieve pixel super-resolution, multiple sub-pixel shifted frames are acquired by translating the light source to different positions using the scanning stage at each illumination angle. In this set-up, the distance between the light source and the sensor (z_1_) is ∼8–10 cm, while the distance between the sample and the image sensor (z_2_) is typically <300–500 µm (which can be increased to >1 mm at the cost of reduced spatial resolution). This geometry, where z_1_>> z_2_, permits recording holograms with unit fringe magnification [15] and therefore the entire active area of the sensor (∼20.5 mm^2^) serves as the imaging field-of-view (FOV). Another important advantage of this geometry is that sub-pixel *hologram shifts* of e.g., ∼0.1–1 µm at the detector plane can be achieved by actually *shifting the light source* by e.g., ∼30–300 µm, which is much easier to achieve without the need for precise positioning. This is indeed a critical enabler to build compact, cost-effective and field-portable computational microscopes that employ pixel super-resolution techniques [20], [21]. The intensity recorded by the sensor-array is a holographically recorded diffraction pattern of the objects, arising from the interference of the un-scattered portion of illumination (reference wave) with the light scattered by the objects (object wave).

The Bayer pattern artefact is clearly observed in the raw images due to the wavelength selectivity of the color filters on the sensor-chip (see e.g., Fig. 1.c). Even if a monochrome sensor was used and full spatial information was retrieved without the Bayer pattern artefact, the recorded lensfree holograms would still be under-sampled due to finite pixel size and the unit-magnification. Therefore, a modified pixel super-resolution algorithm optimized for color sensor-arrays is a critical step to ***i)*** remove the Bayer pattern artefact; and ***ii)*** achieve high-resolution (e.g., ∼350 nm) lensfree imaging beyond what is permitted by the pixel size, that is, the sampling period of the CMOS sensor array.

### Achieving Pixel Super-resolution in Lensfree On-chip Holography using Color Sensors

To solve the under-sampling problem of in-line holograms captured at unit fringe-magnification, we previously utilized pixel super-resolution techniques [19], [20], [37], [38]. This approach had to be modified for color sensors. Using a partially coherent light source with a centre wavelength of 530 nm, maximum response is obtained from the pixels coated with green color filters. Hence, for each unit of the Bayer pattern, only two green channels out of the four available pixels were processed. The data array from two green channels per period (Fig. 2.b) was rotated by 45° degrees so as to obtain a down-sampled hologram ‘*without*’ interpolating the pixel values (see Fig. 2.c). This rotation based approach that eliminates the need for interpolation is rather critical, as interpolation could alter the measured pixels, especially distorting the information in the under-sampled regions that are to be recovered by pixel super-resolution. This down-sampled matrix can be considered as a monochromatic image captured by a virtual sensor, as shown in Fig. 2.d, which is rotated by 45° with respect to the actual image sensor. The *virtual pixel* size (i.e. the pixel size of the virtual CMOS sensor) is equal to the diagonal length of the physical pixels, i.e. ∼1.58 µm in our case. To estimate the sub-pixel shifts between the rotated lower-resolution (LR) images, an iterative gradient method is utilized [38]. This iterative gradient method provides accurate shift estimations, which were validated empirically, even though the rotated pixels are virtual pixels, and half of the area represented by this virtual image is not physically captured (see Fig. 2.b). Then, a pixel super-resolved (SR) hologram is synthesized by optimizing a cost function that minimizes the difference between the estimated SR hologram and the set of measured LR holograms [19], [20], [38]. Through this optimization process, pixel super-resolution decomposes the larger *virtual pixels* of the lower-resolution rotated holograms into effectively much smaller pixels. Therefore, it is critical to have a correct model for spatial light-collection of the *virtual pixels*. This spatial map of light collection for a virtual pixel is provided to the minimization problem as a pixel function. By convolving the ‘estimated’ high-resolution image by this pixel function, then shifting and down sampling the result, a measurement-like image is obtained, which is compared to the actual rotated measurements. In our method that utilizes color sensors, the pixel function was modified to a weighted diamond shape (see Fig. 2.e). With this weighted diamond-shaped pixel function, we obtained superior results compared to using a flat (un-weighted) pixel function.

### Digital Reconstruction of Pixel Super-resolved Holograms to Obtain Lensfree Projection Images

The pixel super-resolved holograms obtained at different illumination angles are reconstructed using the numerical technique reported in Ref. 23. Accordingly, the SR holograms are first multiplied by a tilted plane-wave that represents the reference wave for the corresponding angle of illumination. It is important to note that the tilt angle of this reconstruction wave is not necessarily equal to the physical tilt of the light source due to the refraction of illumination inside the sample chamber. Therefore, the tilt angle, θ_rec_, should be digitally estimated as θ_rec_ = tan^−1^(Δs/z_2_), where Δs is the shift of holograms at a given angle compared to their original position at vertical illumination, and z_2_ is the distance between the detector and the object, which is also estimated digitally using holographic reconstruction. After multiplication by the tilted reconstruction wave, the complex field at the hologram plane is propagated back to the object plane using the angular spectrum approach. In order to remove the twin image noise from the reconstructed images, an iterative phase recovery algorithm is evoked. In this algorithm, by iteratively going back-and-forth between the object and hologram planes, the phase of the hologram can be estimated, and reconstructing this complex field provides a refined lensfree image at the corresponding illumination angle where the twin image noise is suppressed [15].

### Tomographic Reconstruction using Lensfree Projection Images

By performing pixel super-resolution, followed by iterative holographic reconstruction for all angles of illumination (±50°), a set of 51 lensfree projection images is obtained for each orthogonal tilt series. Then, filtered back-projection operation is used to reconstruct 3D images of the sample. To achieve that, we used TomoJ [52], which is a plug-in for the open-source image processing software ImageJ. Accordingly, the projection images are first registered to obtain a common centre-of-rotation using a two-step cross-correlation based algorithm as described in Ref. 23. These projection images are then exported to TomoJ to perform filtered back-projection.

Since the response of the pixels drastically decreases at large angles (e.g. >50–60°), we limited our illumination angles to ±50°. Owing to this limited angular range of each tilt series, isotropic spatial resolution in 3D cannot be achieved. To address this issue dual-axis tomography can be utilized. If a dual-axis tomography scheme is utilized, separate tomograms are initially obtained for each axis. Then, their corresponding tomograms are merged in the Fourier domain [23], [42]. Each single-axis tomogram has missing spatial frequencies, referred to as the “missing wedge”, due to the limited angular range used in back-projection [43]. Dual-axis tomography utilizes the fact that the missing wedge for each set of tomograms will be orthogonal to each other, and the complementary information in these tomograms can reduce the missing wedge in Fourier domain to a “missing pyramid” [43]. Accordingly, a new 3D frequency space is synthesized by assigning the average of the two tomograms to spatial frequencies at which both tomograms have useful data. For frequencies where only one axis provides useful information, no averaging is performed and the value of the corresponding axis is assigned to the new frequency space. An inverse 3D Fourier transformation of the final spatial frequency spectrum provides improved tomograms, which have laterally symmetric point-spread functions as well as improved axial resolution.

### Preparation of the *C. elegans* Sample

Wild-type *C. elegans* nematodes cultured in standard Petri dishes were used in our experiments. A small piece of the culture, containing many worms, was suspended in DI water in a small tube. After 10 minutes, most of the worms swim out of the culture gel into the water. To temporarily immobilize them during image acquisition, levamisole (Tetramisole Hydrochloride 99%, Sigma Aldrich) was added to the tube to obtain a 4 mM solution. Then, 5 µL of the solution was sandwiched between standard cover-slips and placed on the sensor chip for lensfree tomographic imaging.

### Conclusions

We demonstrated lensfree Giga-pixel microscopy and tomography using color image sensors. This platform offers a lateral resolution of ∼350 nm (i.e., a numerical aperture of ∼0.8) over a large imaging area of e.g., 20.5 mm^2^, which is achieved by implementing pixel super-resolution on holographic images captured by state-of-the-art color CMOS sensor-arrays. Further, an axial resolution of ∼2 µm is demonstrated, which can be achieved over a long depth-of-field of ∼0.3 mm. These results correspond to an imaging volume of ∼5 mm^3^ with >150 Billion voxels. Achieving such a large space-bandwidth product within a compact architecture, lensfree optical tomography can provide an important tool for 3D imaging applications in lab-on-a-chip platforms.

## Supporting Information

Movie S1 **Lensfree tomographic imaging of micro-particles over a large field-of-view**. This video shows tomographic on-chip imaging of micro-particles over a large field-of-view.
(MP4)
